# Study on the Electrospinning Fabrication of PCL/CNTs Fiber Membranes and Their Oil–Water Separation Performance

**DOI:** 10.3390/polym17121705

**Published:** 2025-06-19

**Authors:** Desheng Feng, Yanru Li, Yanjun Zheng, Jinlong Chen, Xiaoli Zhang, Kun Li, Junfang Shen, Xiaoqin Guo

**Affiliations:** 1 Henan Key Laboratory of Aeronautical Materials and Application Technology, Zhengzhou University of Aeronautics, Zhengzhou 450046, China; fds@zua.edu.cn (D.F.); yjzheng@zua.edu.cn (Y.Z.); 2 School of Materials Science and Engineering, Zhengzhou University, Zhengzhou 450001, China; 18240508329@163.com (Y.L.); zhangxl@zzu.edu.cn (X.Z.); 3 School of Intelligent Manufacturing, Luoyang Institute of Science and Technology, Luoyang 471023, China; jlchen@lit.edu.cn (J.C.); shen@lit.edu.cn (J.S.); 4 Key Laboratory of Advanced Processing Technology and Application Research for Intelligent Equipment in Luoyang, Luoyang 471023, China

**Keywords:** electrospinning, poly(ε-caprolactone) based membrane, oil–water separation

## Abstract

This study focused on the preparation of poly(ε-caprolactone)/carbon nanotubes (PCL/CNTs) composite membranes via electrospinning technology and investigated their performance in oil–water separation. The effects of varying CNTs contents and spinning parameters on the structure and properties of the membrane materials were systematically studied. A highly uniform diameter distribution of the PCL fiber was achieved by using the dichloromethane/dimethylformamide (DCM/DMF) composite solvent with volume ratio of 7:3, as well as a PCL concentration of ca. 17 wt.%. The optimal electrospinning parameters were identified as an applied voltage of 18 kV and a syringe pump flow rate of 1 mL·h^−1^, which collectively ensured uniform fiber morphology under the specified processing conditions. The critical threshold concentration of CNTs in the composite system was determined to be 1 wt.%, above which the composite fibers exhibit a significant increase in diameter heterogeneity. Both pristine PCL fibrous membranes and PCL/CNTs composite membranes demonstrated excellent and stable oil–water separation performance, with separation efficiencies consistently around 90%. Notably, no significant attenuation in separation efficiency was observed after ten consecutive separation cycles. Furthermore, when incorporating 0.5 wt.% CNTs, the PCL/CNT composite membranes exhibited a 20% increase in separation flux for heavy oils compared to pristine PCL membranes. Additionally, CNTs, as a prototypical class of nanofillers for polymer matrix reinforcement, can potentially enhance the mechanical properties of composite films, thus effectively prolonging their service life.

## 1. Introduction

The primary methods for inducing the alignment of CNTs include electric field induction [1], magnetic field induction [2], and electrospinning [3]. Magnetic field-induced alignment utilizes the magnetic responsiveness of CNTs, enabling their orientation along the direction of an externally applied magnetic field. Electrospinning diverges fundamentally from conventional spinning methodologies by leveraging a high-voltage electrostatic field to ionize polymer solutions or melts, thereby inducing viscoelastic deformation [3]. During this process, a Taylor cone forms at the capillary nozzle tip due to electrostatic charging. When Coulombic repulsion at the droplet interface surpasses the critical threshold of Rayleigh–Plateau instability (i.e., overcoming surface tension), nanoscale polymeric jets are ejected through a whipping instability mechanism [4]. These jets undergo uniaxial elongation under intense electric field gradients, concomitant with rapid solvent volatilization and polymer solidification, culminating in the deposition of ultrafine fibers onto a grounded collector substrate. This technique facilitates the scalable production of fibers with submicron-scale diameters and exceptionally high specific surface areas [5]. The resultant nonwoven architectures exhibit exceptional porosity and percolated fibrous matrices, rendering them ideal candidates for advanced filtration membranes, bioengineered scaffolds, and controlled drug release systems. Aligned fiber assemblies, in particular, demonstrate tailored anisotropic characteristics, such as directional mechanical reinforcement or guided cell proliferation, while offering unparalleled versatility in microstructure–property customization for targeted applications [6,7].

With the acceleration of industrialization, water pollution, particularly chemical oil contamination, has become increasingly severe [8]. Oil–water separation technology holds significant importance in industrial and environmental applications, especially for treating oil–water mixtures containing chemical agents, where separation efficiency is a critical factor. Traditional methods such as sedimentation [9], adsorption [10], and simple filtration are often limited by low efficiency and operational complexity [11]. Consequently, the development of novel, efficient, and renewable oil–water separation materials has emerged as a key research focus [12,13,14]. Electrospinning has gained prominence as an ideal technique for fabricating high-performance separation materials due to its ability to produce fiber membranes with large specific surface areas, tunable microstructures, and high porosity [5]. By adjusting spinning parameters, electrospinning enables precise control over fiber diameter, pore architecture, and alignment patterns. Furthermore, the orientation of fibers can be guided via external electric or magnetic fields, thereby optimizing the performance of the resulting membranes [3].

PCL, a widely used polymer in membrane materials [15,16], exhibits excellent biodegradability, flexibility, and chemical stability [17]. However, its relatively poor hydrophobicity limits its application in oil–water separation, particularly for treating mixtures containing chemical additives. To enhance PCL’s performance and expand its utility in this field, composite modification strategies, such as incorporating CNTs, have been widely adopted [18,19]. CNTs can refine the surface structure of membranes and improve hydrophobicity, thereby enhancing separation efficiency. Notably, during electrospinning, CNTs can align directionally within the PCL matrix, further optimizing the membrane’s surface properties and separation performance [1].

In this study, oriented PCL/CNTs composite fiber membranes were fabricated via electrospinning, and their oil–water separation performance was systematically investigated. The study focused on the effects of varying CNTs concentrations and electrospinning parameters on membrane structure and properties. By comparing membranes prepared under different conditions, the separation efficiency and flux for treating chemically contaminated oil–water mixtures were analyzed. This work provides an environmentally friendly and sustainable solution for industrial wastewater treatment.

## 2. Materials and Methods

### 2.1. Materials

Poly(ε-caprolactone) (PCL, brand Capa 6500) with a melting temperature at around 60 °C, produced by Perstorp Company (Stenungsund, Sweden), was used. Multi-walled carbon nanotubes (MWCNTs, brand TNM2) with a density of 2.1 g/cm^3^ and purity > 95% were produced by Chengdu Organic Chemical Co. Ltd. (Chengdu, China). All organic solvents used in the experiments, such as DMF and dichloromethane, were purchased as analytical grade products from Beijing Yinuokai Technology Co., Ltd. (Beijing, China).

### 2.2. Preparation of the Fibrous Membranes

#### 2.2.1. Preparation of Pure PCL Fibrous Membrane

A precisely formulated solvent (20 mL) was charged into a sealed container, followed by the addition of pre-weighed PCL granules. The above heterogeneous mixture was homogenized using magnetic agitation at 40 °C for 4 h to achieve complete polymer dissolution. To mitigate bubble-induced instabilities during the electrospinning process, the obtained PCL solution was kept for a further 15 min at 40 °C to expel the bubbles produced during the dissolution of PCL granules, thus ensuring rheological equilibrium critical for stable jet formation. The above PCL solution was then used for the preparation of pure PCL fiber membranes, during which the effect of different electrospinning collection devices, different solvent formulas, concentration of PCL in the spinning solution, as well as the parameters of electrospinning on the fiber morphology, were systematically studied.

#### 2.2.2. Preparation of PCL/CNTs Composite Fibrous Membrane

To achieve a homogeneous distribution of CNTs within PCL matrix, a sequential dispersion strategy was implemented. CNTs were first mixed with DMF solvent at predetermined ratios and ultrasonicated in an ice-water bath for 10 min to achieve preliminary dispersion. PCL granules were then added to the mixture and dissolved under constant-temperature magnetic stirring at 40 °C for 4 h, yielding a homogeneous black spinning solution. Similar strategies as those for achieving CNTs dispersion were often used in the preparation of CNTs dispersions [20,21]. To further reduce CNT agglomeration, the above obtained solution was subjected to an additional 1 h of ice-bath ultrasonication, followed by 10 min of standing to eliminate air bubbles. The resulting PCL/CNTs solution was used for the further electrospinning of the composite membranes. During this stage, the synergistic effects of CNTs content and rotation speed of the collector drum on the fiber orientation and the final properties of the composite membranes were investigated.

### 2.3. Characterization

#### 2.3.1. Morphological Characterization

The morphologies of the specimens were observed and recorded with a scanning electron microscope (SEM, FEI Quatan 200, FEI Company, Hillsboro, OR, USA). The prepared samples were cut to the appropriate size and immersed in liquid nitrogen for 1 h. The samples were then removed and quenched quickly to preserve the complete microscopic morphology of the samples. Fracture surfaces were sputtered with gold to provide enhanced conductivity prior to SEM observation.

#### 2.3.2. Wettability Characterization

Wettability analysis was performed using a contact angle analyzer (DSA100, Krüss GmbH, Hamburg, Germany) via the sessile drop method. The contact angles of water (in air) and oil (underwater) on the composite membranes were measured with 0.5 μL droplets. To ensure measurement accuracy, three distinct locations were tested per sample, and the results were averaged.

#### 2.3.3. Porosity Characterization

The porosity of the fiber membranes was calculated using the following formula:

(1) $$\varepsilon =\frac{m_{w}-m_{d}}{\left(m_{w}-m_{d}\right)∕\rho_{0}+m_{d}∕\rho_{p}},$$

where *ε* is the porosity of the fiber membrane (%), *m_w_* is the weight of wet membrane (g), *m_d_* is the weight of dry membrane (g), *ρ*_0_ is the density of water (g/cm^3^), and *ρ*_p_ is the polymer density (1.01 g·cm^−3^ for PCL, determined via water displacement method).

#### 2.3.4. Fiber Orientation Quantification (Hermans Orientation Factor)

The orientation degree of nanofibers was quantitatively analyzed using a statistical approach derived from SEM microstructural characterization. Fifty nanofibers were randomly selected from high-resolution SEM images and their orientation angles relative to the collector drum’s rotation axis were measured using the Directionality plugin in MBF ImageJ 1.54p. The angular distribution data were fitted to the Hermans orientation factor equation:

(2) $$f=\frac{1}{2}(3{cos}^{2}\theta -1),$$

where θ represents the deviation angle between individual fibers. The calculated orientation factor (f) ranges from −0.5 (orthogonal alignment) to 1.0 (ideal uniaxial orientation), with mean values and standard deviations derived from triplicate measurements.

#### 2.3.5. Oil–Water Separation Performance Evaluation

The organic solvent–water mixture with a volume ratio of 1:1 was prepared to test the oil–water separation performance of the film. The oil–water separation capability of the fiber membranes was assessed using a custom-built dead-end filtration system with an effective contact area of 12.56 cm^2^. An oil–water emulsion was prepared by staining n-hexadecane with Sudan III (0.01 wt.%) and water with methylene blue. The emulsion was introduced into the filtration device and allowed to separate under gravitational force. The oil volume pre- and post-separation was quantified to calculate the separation efficiency (*η*) and flux (*J*) using the following equations:

(3) $$\eta =\left(1-\frac{V_{r}}{V_{i}}\right)\times 100\%,$$

(4) $$J=\frac{V}{St},$$

where *V_r_* is the volume after separation, *V_i_* is the initial volume (before separation), *V* is the separated oil volume (L), *S* is the effective membrane area (1.256 × 10⁻^3^ m^2^), and *t* is the separation duration (hour).

## 3. Results and Discussion

### 3.1. Study on the Optimal Electrospinning Parameters for PCL

**Figure 1:**
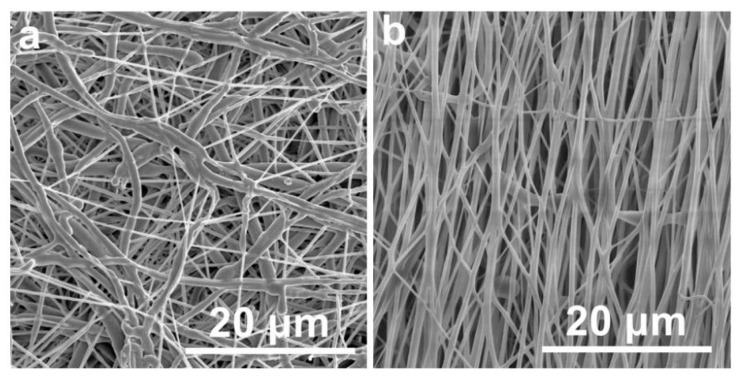
SEM images of randomly arranged fibers collected from the plate collector (**a**) and the oriented arranged fibers collected from the collection roller (**b**).

The structural alignment characteristics of electrospun fiber membranes exhibit significant dependence on collector configuration. This study conducted comparative analyses of stationary planar and rotary cylindrical collectors through systematic experimentation. Comparative analysis of the resultant fiber architectures (Figure 1) revealed distinct morphological variations: planar collectors (Figure 1a) exhibited limited capacity for inducing fiber alignment, whereas cylindrical rotary devices demonstrated enhanced alignment functionality, even under low-speed operation conditions (Figure 1b), which is believed to be induced by the mechanically drawing forces generated during rotational collection process, similar results had been obtained from a previous study [22].

**Figure 2:**
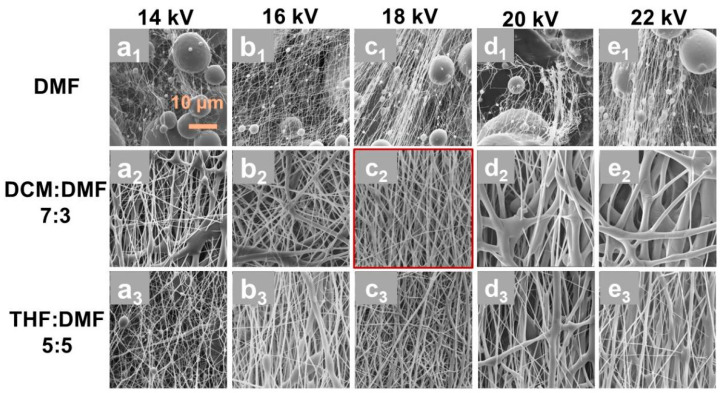
SEM images of PCL fiber membranes prepared using different solvent systems under varying voltage from 14 kV to 22 kV, (**a_1_**–**e_1_**) using solvent system of neat DMF, (**a_2_**–**e_2_**) using solvent system of DCM/DMF with volume ratio of 7:3 and (**a_3_**–**e_3_**) using solvent system of THF/DMF with volume ratio of 5:5.

The solvent used in electrospinning is a critical factor affecting the quality of the resulting fibers. Three different solvent systems, namely pure DMF, DCM/DMF mixed solvent with volume ratio of 7:3, and THF/DMF mixed solvent with volume ratio of 5:5, were used for the electrospinning of PCL, as referred to in other studies [23,24]. The morphological evolution of the prepared PCL fibers using different solvent systems under varying voltages is illustrated in Figure 2.

As shown in Figure 2a_1_–e_1_, pure DMF consistently produced fibers with excessive bead formation and lower fiber production efficiency across all voltages, which is mainly caused by the high resistivity and poor volatility of pure DMF, leading to insufficient traction force and solvent retention effects in the electrostatic field during the electrospinning process. The introduction of co-solvents can effectively improve the stability of the jet. DCM and THF are commonly used co-solvents in electrospinning. When DCM/DMF mixed solvent was used for the electrospinning of PCL, as shown in Figure 2b_2_–e_2_, bead-free fiber formation with narrowed diameter distribution was achieved at optimized voltages, which is believed to be caused by the superior volatility and higher dielectric constant of DCM. In contrast, the THF/DMF system exhibited residual bead structures and broader diameter dispersion, attributable to the reduced volatility of THF compared to DCM.

Meanwhile, through comparative analysis of the PCL fiber morphology fabricated under varying applied voltages from 14 kV to 22 kV, it was observed that the employing of an electrospinning voltage of 18 kV yielded PCL fibers with uniform diameter distribution and partial molecular orientation. However, the electrospinning voltage can significantly influence fiber formation dynamics [25,26,27]. Specifically, low electrospinning voltage may induce inadequate jet formation due to insufficient electric field force, resulting in droplet deposition prior to solvent evaporation [25], as shown in Figure 2a_2_,b_2_. Conversely, excessive voltage promoted jet instability, ultimately leading to jet breakage during the electrospinning process, as shown in Figure 2d_2_,e_2_.

**Figure 3:**
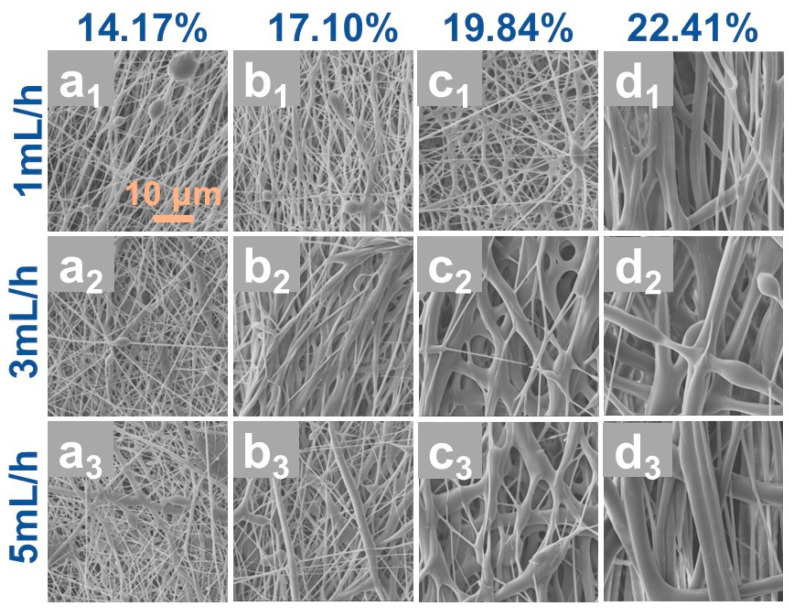
SEM images of PCL fiber membranes prepared at different propulsion speeds under different PCL concentrations of from 14.17 wt.% to 22.41 wt.%, (**a_1_**–**d_1_**) propulsion speed of 1 mL/h, (**a_2_**–**d_2_**) propulsion speed of 3mL/h, and (**a_3_**–**d_3_**) propulsion speed of 5 mL/h.

Based on the aforementioned findings, an applied voltage of 18 kV combined with a DCM/DMF solvent system was selected (as indicated in Figure 2c_2_) to investigate the effects of feed rate and PCL concentration on the morphological evolution of electrospun fibers, as summarized in Figure 3. The results demonstrate a positive correlation between PCL concentration and fiber diameter (Figure 3a–d). At a PCL concentration of 17.1 wt.% (5 g PCL into 20 mL DCM/DMF), the resultant fibers exhibited smaller diameters with enhanced uniformity. Conversely, reducing the PCL concentration to 14.17 wt.% (4 g PCL into 20 mL DCM/DMF) induced the formation of distinct bead structures (Figure 3a_1_–a_3_). This phenomenon arises from the inherently low molecular weight of PCL, where insufficient polymer chain entanglement at hypo-concentrated conditions destabilizes the electrospinning jet via reduced solution viscosity, thereby promoting bead defects through Rayleigh instability. Elevating the PCL concentration to 22.41 wt.% (7 g PCL into 20 mL DCM/DMF) eliminated bead formation; however, the excessive viscoelastic resistance of the hyper-concentrated solution hindered effective jet attenuation under the applied electric field, yielding oversized fibers with diminished specific surface area (Figure 3d_1_–d_3_), which compromises the oil–water separation efficiency of the resultant membranes.

Furthermore, the syringe feed rate critically influenced fiber morphology [28,29]. Moderate increases in feed rate mitigated bead formation while inducing a gradual diameter increment. Nevertheless, excessively high feed rates (e.g., 5 mL/h) produced fibers with non-uniform diameters and pronounced polydispersity. Consequently, optimizing both PCL concentration and feed rate is imperative for fabricating high-performance PCL/CNTs composite membranes. Notably, the incorporation of CNTs significantly elevates solution viscosity. To counteract this effect, reduced PCL concentrations (17.1 wt.%) and lower feed rates (1 mL/h) were adopted for subsequent PCL/CNTs composite membrane fabrication, ensuring balanced processability and structural homogeneity.

### 3.2. Influence of CNTs Content on the Morphology of Fiber Membranes

Based on the above research results of pure PCL fiber membranes, in this section, the same preparation process was adopted to prepare PCL/CNTs composite fiber membranes, namely, using a cylindrical collection roller, a spinning voltage of 18 kV, a dcm/dmf mixed solvent system, a PCL concentration of 17.1 wt.%, and a propulsion speed of 1 mL/h. On this basis, the effects of CNTs content and the rotational speed of the collection roller on the structure and properties of the prepared composite fiber membrane were studied.

**Figure 4:**
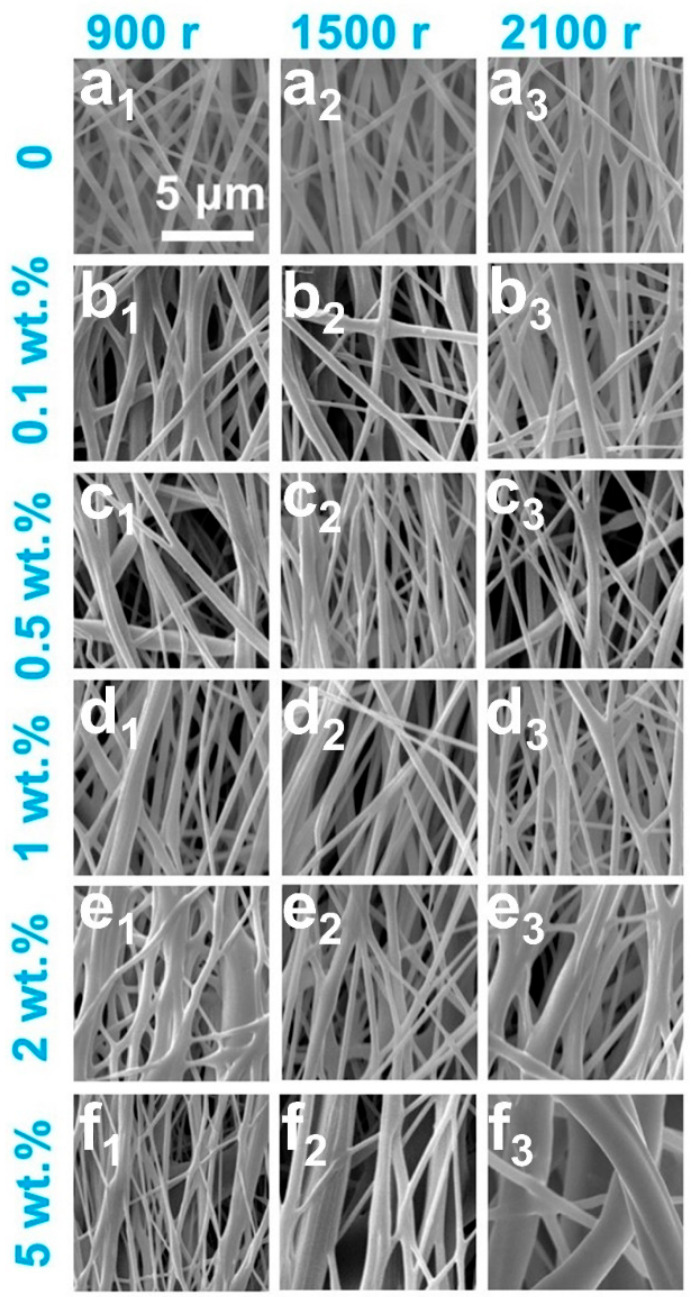
SEM images of PCL/CNTs composite fiber membranes prepared using different CNTs concentrations under different collection roller speeds ranging from 900 r to 2100 r, (**a_1_**–**a_3_**) pure PCL, (**b_1_**–**b_3_**) 0.1 wt.% CNTs, (**c_1_**-**c_3_**) 0.5 wt.% CNTs, (**d_1_**–**d_3_**) 1.0 wt.% CNTs, (**e_1_**–**e_3_**) 2.0 wt.% CNT and (**f_1_**–**f_3_**) 5.0 wt.% CNTs.

**Figure 5:**
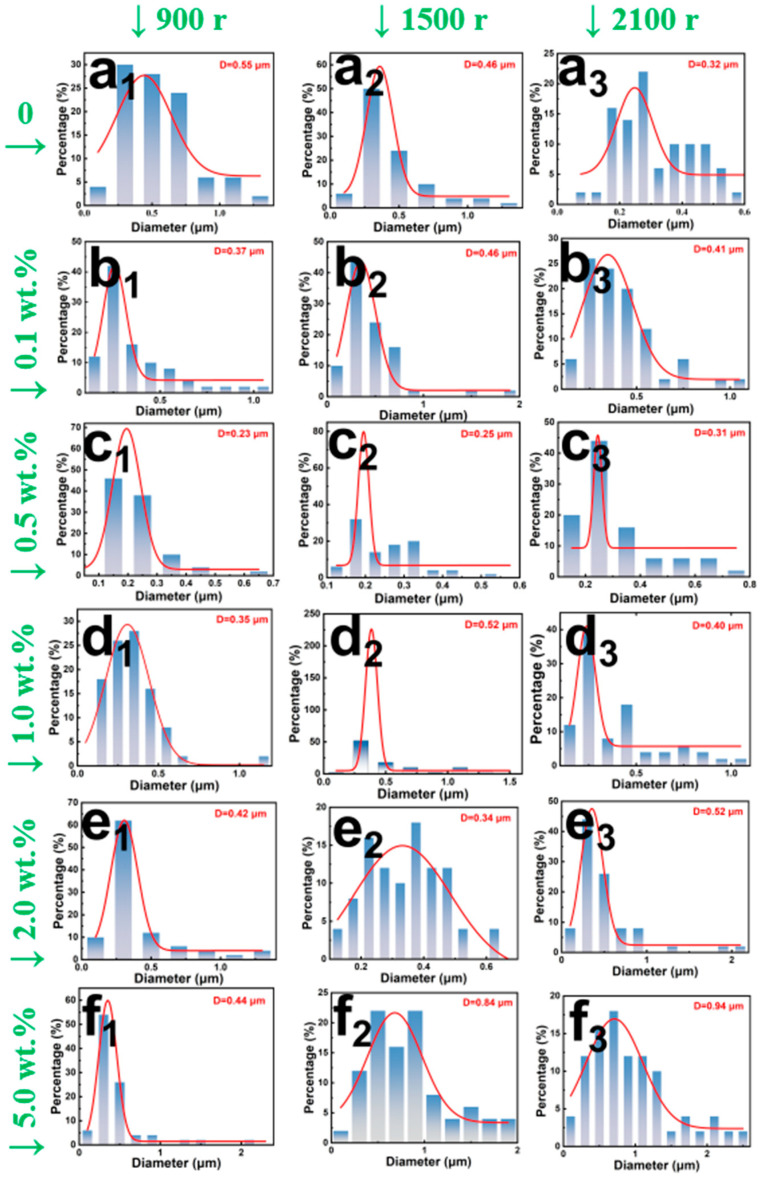
Diameter statistics PCL/CNTs composite fibers prepared under different collection roller speeds ranging from 900 r to 2100 r and CNTs concentrations, (**a_1_**–**a_3_**) pure PCL, (**b_1_**–**b_3_**) 0.1 wt.% CNTs, (**c_1_**–**c_3_**) 0.5 wt.% CNTs, (**d_1_**–**d_3_**) 1.0 wt.% CNTs, (**e_1_**–**e_3_**) 2.0 wt.% CNT and (**f_1_**–**f_3_**) 5.0 wt.% CNTs.

In electrospinning processes, precise control over filler content is critical to maintaining fiber continuity. Excessive introduction of CNTs could induce a marked increase in the viscosity of the polymeric solution, thereby impeding jet elongation and ultimately resulting in heterogeneous fiber diameter distributions and bead formation [29,30]. Furthermore, the dispersion efficacy of CNTs within PCL matrix is inherently limited under high-viscosity conditions, as ultrasonic treatment fails to fully mitigate agglomeration, which may precipitate nozzle blockages. Consequently, the upper threshold for CNTs content was empirically established at 5 wt.% in this investigation. SEM images of PCL/CNTs composite fiber membranes prepared under different collection roller speeds and CNTs concentrations are shown in Figure 4, which revealed a pronounced correlation between CNTs loading and fiber diameter heterogeneity. Quantitative analysis of 50 fiber diameters via MBF ImageJ software, coupled with Origin2021-generated histograms, as shown in Figure 5, demonstrated that CNTs loadings exceeding 1 wt.% significantly amplified diameter polydispersity. When the CNTs concentration is as high as 5 wt.%, a precipitous rise in solution viscosity exacerbated dispersion challenges, leading to the partial exposure of CNTs on fiber surfaces and conspicuous agglomerate formation. These morphological irregularities not only undermine structural homogeneity but also potentially degrade the mechanical robustness of the membranes.

Fiber alignment was governed by the synergistic effect between collector rotation speed and jet stretching dynamics. Theoretical frameworks posit that uniaxial alignment is achievable when the collector speed aligns with the fiber deposition rate [31]; however, excessive rotation could also induce fiber curvature and diminished orientation. Experimental observations indicated that at a rotational speed of 900 rpm, insufficient electrostatic stretching yielded fibers with elevated diameters ranging from 0.2 um to 0.3 um. Conversely, at the highest rotation speed of 2100 rpm, hyper-stretching broadened the diameter distribution and induced mechanical failure in fibers surpassing the tensile limits of PCL.

The orientation parameter was calculated via the Hermans equation and summarized in Table 1, which indicated that all fibers exhibited an orientation degree clustered around 0.80. Notably, membranes with CNTs loadings of 0 wt.% and 0.1 wt.% attained peak orientation values of 0.90, suggesting minimal disruption to PCL molecular chain alignment at low filler concentrations. Incremental increases in CNTs loading elevated solution viscosity, thereby impeding stretching efficiency and marginally reducing orientation.

### 3.3. Impact of CNTs Content on Hydrophobic Properties of Different Fiber Membranes

**Figure 6:**
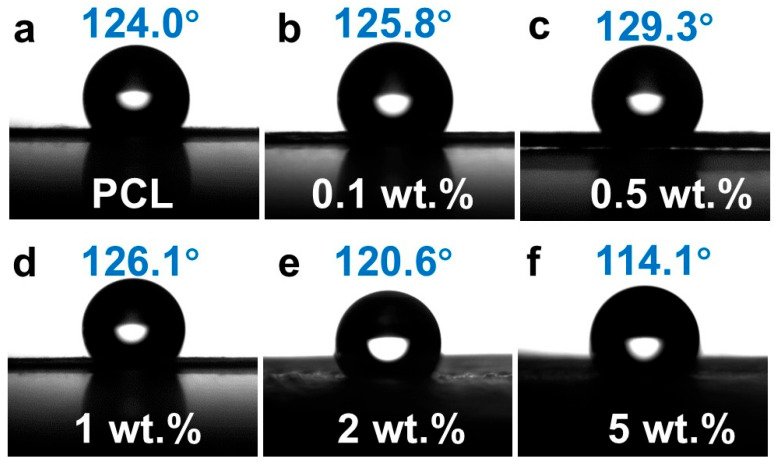
The water contact angle of PCL/CNTs composite fiber membranes with different CNTs contents, (**a**) pure PCL, (**b**) 0.1 wt.% CNTs, (**c**) 0.5 wt.% CNTs, (**d**) 1.0 wt.% CNTs, (**e**) 2.0 wt.% CNT and (**f**) 5.0 wt.% CNTs.

**Figure 7:**
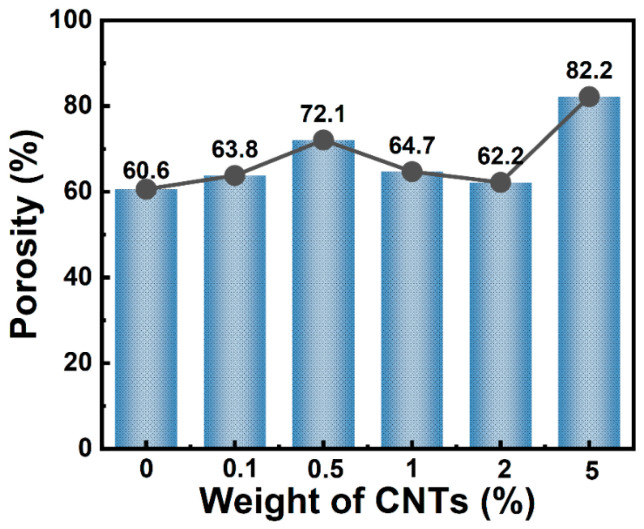
Porosity of PCL/CNTs composite fiber membranes with different CNTs loadings.

Wettability analyses were conducted to elucidate the role of CNTs in modulating surface hydrophobicity on the fiber membranes prepared using rotation speed of 1500 rpm. Water contact angle (WCA) measurements across three randomly selected regions per specimen revealed a non-monotonic trend: WCA initially ascended from 124.0° for the pristine PCL fiber membrane to 129.3° for PCL/CNTs composite membrane with CNTs content of 0.5 wt.%, followed by a decline to 114.1° when CNTs content increased to 5.0 wt.%, as shown in Figure 6. The apex in hydrophobicity at 0.5 wt% CNTs aligns with the Cassie–Baxter regime, wherein enhanced surface roughness promotes air pocket entrapment [32,33]. While, when CNTs content is above 1.0 wt.%, agglomerate-induced heterogeneity in surface topography generated hydrophilic domains, thereby diminishing WCA. The intrinsic hydrophobicity of PCL arises from its nonpolar alkyl backbone, which repels polar water molecules, while the weakly polar ester moieties (-COO-) contribute negligibly. CNTs integration modulates wettability by altering surface topology and pore architecture [34,35], as evidenced by porosity analyses in Figure 7.

### 3.4. Porosity Evolution with CNTs Incorporation in Fiber Membranes

Porosity quantification via Equation (1) was shown in Figure 7, which demonstrated that pristine PCL membranes exhibited a porosity of 60.6%; while, at the 0.1 wt.% loading of CNTs, the porosity of PCL/CNTs composite fiber membrane marginally increased to 63.8%. A notable surge to 72.1% porosity at 0.5 wt.% loading of CNTs was attributed to microvoid nucleation stemming from poor CNTs-PCL interfacial compatibility. However, further increases in CNTs loading intensified agglomeration, reducing porosity to 62.2%. Intriguingly, when CNTs loading is as high as 5.0 wt.%, the porosity of PCL/CNTs composite fiber membrane rebounded to 82.2%, a phenomenon ascribed to macropore formation via CNTs aggregates and increased fiber diameter, which collectively reduce packing density.

### 3.5. Evaluation of Oil–Water Separation Efficacy

**Figure 8:**
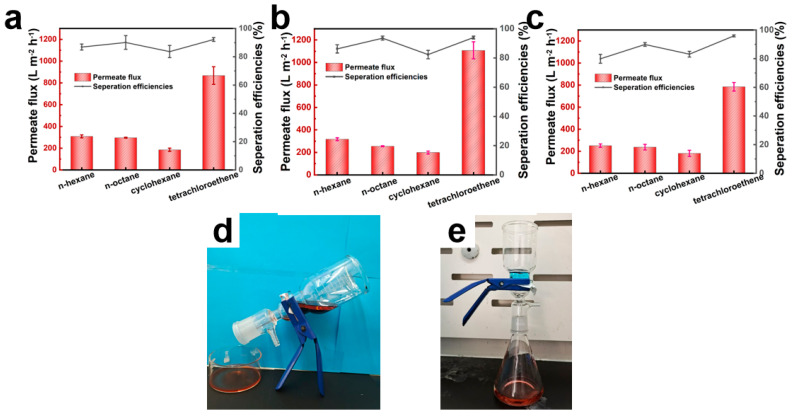
Oil-water separation performance of the fiber membranes with different CNTs content, (**a**) 0 wt.%, (**b**) 0.5 wt.%, (**c**) 1.0 wt.%, (**d**) schematic diagram of light oil separation device, (**e**) schematic diagram of heavy oil separation device.

**Figure 9:**
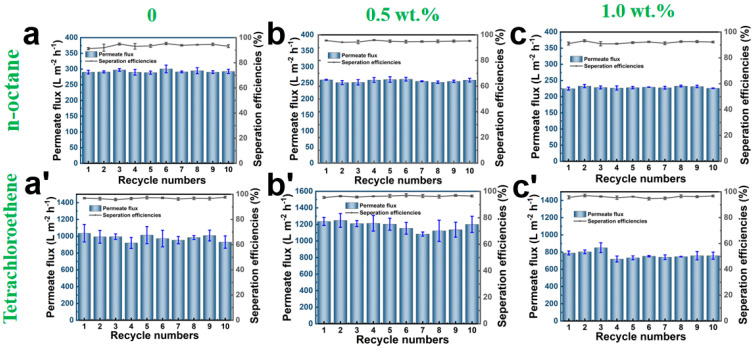
The filtration cycle stability of fiber membranes with different CNTs content for n-octane (**a**–**c**) and perchloroethylene (**a’**–**c’**), with CNTs contents of 0, 0.5 wt.% and 1.0 wt.%, respectively.

Two distinct separation devices were employed to separate oil–water mixtures of light and heavy oils, as illustrated in Figure 8d and Figure 8e, respectively. The pure and composite fiber membranes with 0 wt.%, 0.5 wt.%, and 1.0 wt.% loading of CNTs were subjected to oil–water separation experiments. The results are shown in Figure 8 and Figure 9. The PCL/CNTs composite fiber membrane with 0.5 wt.% loading of CNTs exhibited maximal flux, outperforming pristine PCL, while the composite membrane with 1.0 wt.% loading of CNTs displayed the lowest performance. Tetrachloroethylene demonstrated superior flux relative to lighter oils (e.g., n-hexane, n-octane), attributable to gravitational dominance in pore penetration dynamics. The composite membrane with 0.5 wt.% loading of CNTs achieved optimal separation efficiency, which is above 90%, owing to synergistic effects of enhanced hydrophobicity and uniform pore distribution.

Cyclic durability tests in Figure 9 revealed stable separation efficiency over 10 cycles, with pristine PCL exhibiting the highest tetrachloroethylene flux of 1100 L·m^−2^·h^−1^. The separation flux decreased to 1000 L·m^−2^·h^−1^ for composite membranes with CNTs loading of 0.5 wt.% and 900 L·m^−2^·h^−1^ when CNTs loading increased to 1.0 wt.% CNTs, which is believed to be caused by the agglomerate-induced pore constriction of the composite fibers.

Nevertheless, while maintaining high separation efficiency, the separation flux of the composite membrane prepared in this work significantly surpasses values reported in many studies. As summarized in Table 2, which compiled research data on polymer-based composite membranes for oil–water separation, the composite membrane fabricated in this study demonstrated over 90% separation efficiency with a membrane thickness of only ca. 150 μm, which is slightly lower than the literature values. However, its separation flux reached approximately 1000 L·m^−2^·h^−1^, far exceeding the data reported in the referenced literature by a large margin.

## 4. Conclusions

In this study, we investigated the structure–property interdependencies of aligned PCL/CNTs composite membranes fabricated via electrospinning, with emphasis on CNTs loading and process parameter optimization. The optimal parameters for prepared PCL-based composite fiber membranes were identified as follows: using the DCM/DMF mixed solvent, PCL concentration of ca. 17 wt.%, applied electrospinning voltage of 18 kV, as well as feed rate of 1.0 mL/h, yielded composite fibers with narrow diameter distributions. Furthermore, the synergistic effects of CNTs content and rotational speed of the collection roller on the morphology of the prepared PCL/CNTs composite fiber membranes were systematically studied, indicating that CNTs with a loading of 0.5 wt.% preserved fiber diameter stability and achieved the best results for both flux and hydrophobicity, whereas CNTs loading surpassing 1.0 wt.% induced diameter escalation via agglomeration. Finally, the PCL/CNTs composite fiber membrane exhibited an optimal oil–water separation flux of and 1000 L·m^−2^·h^−1^ and stable cyclic separation efficiency of above 90%, surpassing both pristine PCL and other PCL/CNTs composite fiber membranes.

## Figures and Tables

**Table 1:** Orientation parameter of PCL/CNTs fibers with varying CNTs content.

	0	0.1 wt.%	0.5 wt.%	1.0 wt.%	2.0 wt.%	5.0 wt.%
900 rpm	0.80	0.82	0.84	0.90	0.76	0.77
1500 rpm	0.89	0.92	0.80	0.83	0.88	0.79
2100 rpm	0.91	0.84	0.74	0.80	0.80	0.80

**Table 2:** Oil–water separation performance of the polymer-based membranes.

Material	Permeation Flux (LMH)	Separation Efficiency (%)	Type of Emulsion	Number of Cycles	Reference
PCL/CNTs	1000	>90	O/W	10	This work
PVC/SiO_2_	358	95	O/W	10	[36]
PVDF/PVA	318	99.9	O/W	30	[37]
PVDF/TiO_2_	400	99.7	O/W	5	[38]
PAI/HFPS	440	99.9	O/W	18	[39]
PVDF/PDA/PEI	233	85	O/W	3	[40]
PAN-PA6/PANI	326	97.8	O/W	5	[41]
PAN/MOF	380	99.97	O/W	10	[42]
PVDF/ESP	229	99.5	O/W	10	[43]
PES/SiO_2_-g-PMAA	143	98	O/W	-	[44]
PMMA/PEI/PVDF	90	99.3	O/W	10	[45]
PVDF/ZnO	1.11	93	O/W	-	[46]

## Data Availability

Data will be made available on request.
